# Preparation and Characterization of Co-Diamond Composite Coatings Obtained in a Single-Step Hybrid Electrophoretic Deposition Process

**DOI:** 10.3390/ma18061294

**Published:** 2025-03-15

**Authors:** Diana Uțu, Roxana Muntean, Iasmina-Mădălina Anghel (Petculescu), Iosif Hulka, Ion-Dragoș Uțu

**Affiliations:** 1 Department of Pharmacology-Pharmacotherapy, Faculty of Pharmacy, Victor Babes University of Medicine and Pharmacy Timisoara, Eftimie Murgu Sq. 2, 300041 Timisoara, Romania; diana.utu@umft.ro; 2 Department of Materials and Fabrication Engineering, Politehnica University Timisoara, Bulevardul Mihai Viteazul nr.1, 300222 Timisoara, Romania; iasmina.anghel@student.upt.ro; 3 Research Institute for Renewable Energy, Politehnica University of Timisoara, G. Musicescu 138, 300501 Timisoara, Romania; iosif.hulka@upt.ro

**Keywords:** electrodeposition, Co-based composite coatings, corrosion resistance, wear properties

## Abstract

The electrochemical co-deposition of various hard particles with metals or metal alloys has been recently studied, especially for developing wear-resistant coatings. In the current work, pure cobalt and cobalt–diamond composite coatings were electrochemically deposited onto a low-alloy steel substrate and further investigated in terms of microstructure, corrosion behavior, and tribological characteristics. The electrodeposition process was carried out using direct current, from an additive-free electrolyte containing 300 g L^−1^ CoSO_4_, 50 g L^−1^ CoCl_2,_ and 30 g L^−1^ H_3_BO_3_ with and without diamond particles. Scanning electron microscopy (SEM) combined with energy-dispersive X-ray spectroscopy (EDS) was used for the microstructural characterization correlated with the chemical composition identification of the resulting coatings. The pure Co coatings showed a dense microstructure with a nodular morphology. In contrast, for the Co-diamond composite coatings, more elongated grains were observed containing a uniform distribution of the reinforcing diamond particles. The corrosion resistance was evaluated by potentiostatic polarization measurements in 3.5 wt.% NaCl solution, while the sliding wear resistance was assessed using the ball-on-disk testing method. The experimental results demonstrated that incorporating diamond particles into the cobalt deposition electrolyte positively impacted the tribological performance of the resulting composite coatings without significantly affecting the corrosion properties. Both cobalt and the composite coatings demonstrated substantially superior wear characteristics and corrosion resistance compared to the steel substrate.

## 1. Introduction

According to the available statistics, the economic losses caused by corrosion and friction-related phenomena, in industrialized countries, represent between 5 and 10% of the gross domestic product [1,2]. Hence, worldwide, several corrosion and wear mitigation strategies are taken into consideration to reduce the negative effects that are often associated with disasters, severe failure of equipment, environmental side effects, or increased maintenance costs [1]. One practical method for reducing superficial damage in engineering applications is to modify the surfaces of materials without changing their bulk properties [3]. Since various applications in the technical field require high resistance to aggressive conditions, different types of surface modifications, in the form of coatings, layers, or films, have been recently proposed to serve the increasingly exigent industrial expectations. Functional coatings are essential in a wide range of applications, starting from the food industry, medicine, aerospace, or agriculture [4]. The recent progress in coating technology, correlated with the economic impact generated by the mitigation of damaging effects, has led to a more intense interest of scientists in discovering novel coating systems with improved performances and innovative deposition techniques adapted to actual industrial needs [5,6,7]. Regardless of the class of materials they belong to, or the thicknesses they exhibit, generally, the coatings should provide important characteristics like resistance to corrosion, erosion, wear, adhesion to the substrate, mechanical strength, and/or oxidation resistance, without affecting the main function of the component. Furthermore, it has been demonstrated that some of the mentioned properties can be additionally improved by the incorporation of hard phases in metallic coatings [8]. In these circumstances, the coatings belong to the class of composite materials, consisting of a soft and ductile component as a matrix, and a harder, wear-resistant reinforcer usually in the form of particles or fibers. Composite coatings comprising refractory ceramic particles usually exhibit good wear properties [9]. Furthermore, the mechanical properties, as well as corrosion behavior, can be greatly improved in such composites compared to pure metallic coatings. Commonly, the strengthening effect in the metal matrix composites may be assigned to a set of long-range and short-range forces occurring from the presence of the second-phase particles [10]. The particle interaction in the composite coating was found to be important in dislocation due to the different elastic properties of the two constituents and the local resistance of the particles as obstacles to the dislocation motion. The improved properties mainly depend on the ceramic particle content and the nature of the metal matrix. Moreover, by embedding ceramic particles into a metallic matrix, properties such as self-lubrication [11] and corrosion resistance [12] have also been significantly improved [13].

Recent advancements on this topic include the development of nanocomposite coatings, which offer even greater performance by incorporating nanoscale reinforcements [14]. Additionally, functionally graded materials are being explored to create coatings with varying properties across their thickness, optimizing them for specific applications. Although it is possible to obtain composite coatings by various deposition techniques [15,16,17,18,19,20], like laser cladding [21], thermal spraying [22], laser melt injection [23], brazing [24] and electrospark coating deposition [25], the electrodeposition process is preferred in some cases due to the several advantages provided, such as ease of application, reproducibility, low cost, and high efficiency compared to other coating technologies [26]. Particularly, the process applied for depositing charged ceramic particles from an aqueous-based colloidal electrolyte onto a conductive substrate is called electrophoretic deposition (EPD). It is usually employed to deposit high-purity ceramic coatings for biomedical applications and is commonly followed by a sintering process [27]. Recently, this deposition process was adapted to co-deposit the ceramic particles along with metals and alloys, in order to form composite coatings. In this case, the deposition can be considered a hybrid one, combining the advantages of the electrolytic and the electrophoretic deposition in one step. The main parameters of the suspension that may influence the morphology of the resulting coating include particle size, electrical conductivity, viscosity, or stability of the suspension. Deposition parameters, like duration, applied voltage, deposition type and conductivity of the substrate, also have significant effects on the process.

Among the metals that benefit from this deposition technology is cobalt (Co) [28], which is known for its remarkable properties, like hardness and high melting point, excellent resistance to corrosion and wear, as well as exceptional mechanical, electrical, magnetic, and chemical properties [8]. In this context, Co is recommended for replacing hard chromium coatings, for the development of coatings with practical applicability in gas turbines, automotive, medical, chemical, and petroleum industries, as well as chemical catalysis [29]. Considerable efforts have also been made to adopt environmentally friendly processes, to address the challenges related to the high cost, health, and safety concerns associated with cobalt-based coatings [30,31]. Additionally, Co-based composite coatings are relatively less expensive compared to other composite coatings such as nickel-based composites, epoxy/alumina composites, or copper-based composites [8]. Nowadays, Co-based composite coatings are increasingly vital for enhancing the durability and performance of industrial components subjected to extreme conditions. These coatings, composed of a Co matrix with embedded reinforcing particles like carbides [32], oxides [33], polymers [34], or graphene/carbon fibers [35], are known for their exceptional wear behavior, corrosion resistance, and stability at high temperatures. A series of studies have explored the fabrication and properties of Co-based composite coatings deposited by laser cladding technique [36,37,38]. Weng [36] and Yan [37] both found that the addition of CeO_2_ and CaF_2_ particles, respectively, improved the microhardness and wear resistance of the Co-based coatings. Weng et al. [38] further enhanced these properties by adding Ti_5_Si_3_ and TiC. Toosinezhad et al. [39] developed composite layers based on pure cobalt and cobalt/graphene produced by electrodeposition technique and found out that both types of coatings presented a higher hardness compared to the substrate material, while the cobalt/graphene microhardness was around 3 times higher than the pure Co coating. Ramesh Bapu and Thiruchelvam [40] found that Co-BN composites exhibited improved corrosion resistance in NaCl and HCl solutions compared to pure cobalt. They established that factors affecting particle incorporation in these composites include bath chemical composition, current density, pH, and temperature. Toosinezhad et al. [41] developed Co-graphene composite coatings deposited on a St37 steel substrate which exhibited increased hardness, better tribological behavior, and improved corrosion resistance in NaCl solution compared with the base material.

Based on the proven potential of Co composite coatings, the current work aims to evaluate the possibility of obtaining Co-diamond composites in a single-step hybrid electrophoretic deposition. The effect of the diamond particle incorporation into the electrochemically deposited cobalt coatings is evaluated, analyzing the most significant characteristics like sliding wear and corrosion behavior. The results were compared with a pure cobalt coating obtained in similar deposition conditions, as well as with the low-alloy steel used as substrate.

## 2. Materials and Methods

The substrate material employed as support for the cathodic electrodeposition of the Co-based coatings was a commercial S235 structural carbon steel cut into rectangular coupons of 20 mm × 20 mm × 5 mm. The chemical composition of the S235 steel was established using an optical emission spectrometer (OES) (Thermo Electron Corporation Genesys, Thermo Fisher Scientific, Waltham, MA, USA), and the values are presented in Table 1. Before deposition, the surface of the steel substrate was mechanically polished using various grades of abrasive paper to achieve a mirror-like metallic surface with a roughness of *R_a_* = 0.2 µm, followed by ultrasonic cleaning in distilled water and ethanol. Subsequently, after drying, the surface was soaked in 2M HCl solution at 25 °C for 15 s, to remove the residual oxides, followed by an additional rinse with distilled water.

To improve the adhesion of the Co coating to the steel substrate, an intermediate thin nickel layer (5 μm) was first electrochemically deposited onto the steel surface, from a typical Watts solution. The electrochemical deposition of both Ni and Co was performed in a two-electrode cell configuration, with continuous stirring, with the aid of a programmable direct current power supply (Voltcraft PPS-16005 36 V, 10 A, Conrad Electronic SE, Hirschau, Germany). The deposition electrolytes were prepared by dissolving the commercially available chemical reagents in distilled water. The composition of the Ni and Co electrolyte, as well as the working conditions, are presented in Table 2. Additionally, to produce the Co composite coatings, diamond particles (0.5 μm) were added to the Co-based electrolyte, generating a 2.5 g L^−1^ concentrated suspension. The diamond particles are therefore co-deposited in the Co coating during the electrochemical deposition process. The deposition rate and coating thickness were controlled by varying the applied current and the deposition time. While the role of the Ni and Co salts in the deposition electrolyte is as a source of metal ions, the H_3_BO_3_ is added to stabilize the pH during the deposition, near the electrode surface. The working conditions were experimentally adjusted to obtain a uniform Co-based coating, with a thickness of around 50 μm. The morphology and microstructure of the electroplated samples were analyzed using an FEI Quanta™ FEG 250 (FEI Quanta™, Hillsboro, OR, USA) scanning electron microscope (SEM) equipped with energy-dispersive spectroscopy analyzer (EDS) for chemical composition identification. The roughness of the surface before the electrochemical deposition was evaluated with a Mitutoyo SJ-201 portable surface tester (Mitutoyo, Aurora, IL, USA). The thickness of the coatings was checked in cross-section, using SEM analysis.

The corrosion resistance of the Co-based coatings with and without diamond particles was investigated by potentiodynamic polarization, in a 3.5 wt.% NaCl solution, and compared to the S235 steel substrate, as reference. All electrochemical measurements were carried out in a three-electrode corrosion cell connected to a potentiostat/galvanostat (Biologic SP 150, Biologic, France). The working electrode consisted of the Co-based coated samples (1 cm^2^ exposed surface), the reference was a Ag/AgCl (3M NaCl) electrode positioned near the working electrode via a Luggin capillary, and the counter electrode was a Pt gauze. The potentiodynamic polarization curves were recorded at normal temperature with a scan rate of 0.16 mV s^−1^ by sweeping the potential between ± 500 mV and the open circuit potential (OCP). The main corrosion parameters were evaluated from the obtained polarization curves using the Tafel extrapolation method in the linear region of the anodic and cathodic branches and compared to the steel substrate. The microhardness of the coatings and substrate was evaluated on the polished cross-sectioned sample using a Vickers indenter (ZHVμ device from Zwick/Roell, Ulm, Germany). The reported values (HV0.3) represent the average of five measurements, each performed under a vertical load of 30 g for a duration of 15 s. The sliding wear behavior of the steel substrate and the Co-based coatings was evaluated using the ball-on-disc method, according to the ASTM standard G99 [42]. The investigations were performed with the aid of a TR-20 Tribometer, (Ducom-Materials Characterisation Systems), under dry sliding conditions and ambient temperature, setting a normal load of 5 N, sliding rate of 100 rot min^−1^, wear track diameter D = 10 mm, test time of 100 min, and a total sliding distance 314 m. The counterbody selected for the tests was a 100Cr6 steel ball with a 6 mm diameter. During the tests, the coefficient of friction (COF) variation with the distance was automatically recorded. The wear track profile after the tests was subsequently analyzed, and the wear rates were estimated. For each type of sample, three measurements were performed in order to ensure the reproducibility of the results.

## 3. Results and Discussion

### 3.1. Microstructure and Chemical Composition

**Figure 1:**
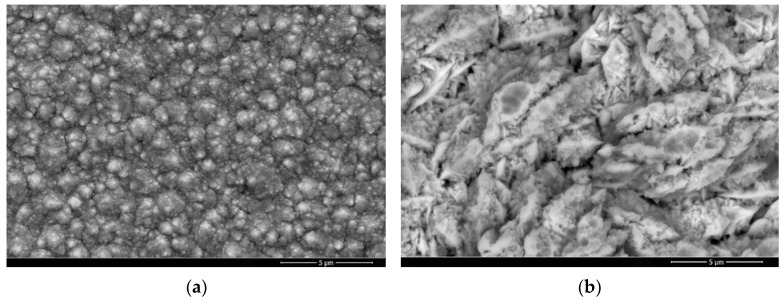
SEM surface micrographs of the (**a**) Co coating and (**b**) Co + diamond particle composite coating.

Figure 1 presents the SEM surface micrographs of the electroplated Co-based coatings with and without diamond particles. In both cases, the coatings are dense, compact, and bright, and no micro-cracks were observed in the structure. Although the electrodeposition of Co from an aqueous solution occurs with an intense hydrogen evolution reaction, no pits or defects associated with this phenomenon can be observed. For the pure Co coating deposited at 50 mA cm^−2^ current density (Figure 1a), the morphology exhibits fine, homogeneous nodular grains, with a uniform size distribution. Numerous studies reported that the usual surface morphology of the electrodeposited Co coatings exhibits nodular or granular structures [43]. However, the addition of diamond particles into the Co electrolyte, correlated with a lower applied deposition current density (25 mA cm^−2^), alters the growth mechanism, shape, and size of the Co grains. The diamond particles may enter the Co lattice gaps and generate dislocations, which lead to changes in the grain morphology of the composite coating. In this case, more elongated features of the microstructure were observed, while the morphology became coarser, characterized by larger, randomly oriented grains (Figure 1b). This observed surface morphology is consistent with literature reports on alloy and composite coatings. Similar behavior was observed in the case of cobalt films deposited onto a copper substrate, in comparable working conditions [44]. Furthermore, it has been shown that when a higher current density is applied, a more reduced incorporation of diamond particles is achieved [16].

**Figure 2:**
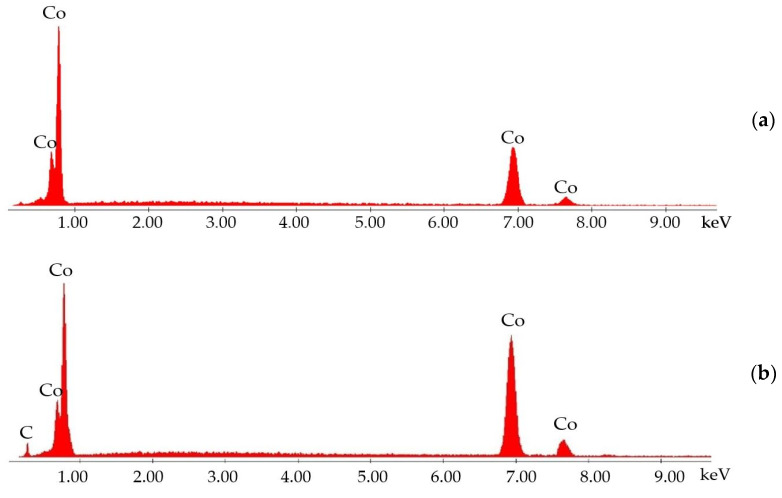
EDS spectra of the (**a**) Co coating and (**b**) Co + diamond particle composite coating.

The EDS spectra (Figure 2) reveal the chemical composition of the coatings, composed only of Co, as the main constituent, without any chloride or oxygen contamination detectable through EDS. In the case of Co composite coating, the rise of the C signal confirms the presence of diamond particles embedded in the Co matrix (Figure 2b).

**Figure 3:**
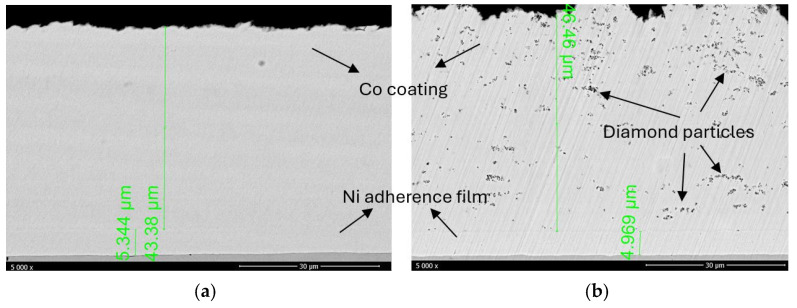
Cross-section SEM micrographs of the (**a**) Co coating and (**b**) Co + diamond particle composite coating.

The polished cross-sectional view (Figure 3) further demonstrates that the coatings are free from defects such as pores or cracks along the whole thickness. The Ni interlayer is poorly visible in the SEM micrographs since the contrast for both chemical elements, Co and Ni, is similar. The interface between the substrate and the Ni film shows no defects, inclusions, or signs of coating delamination. In the case of the Ni layer, the coating thickness was approximately 5 ± 1 µm. Notably, the SEM micrograph of the composite coating (Figure 3b) highlights the successful incorporation of diamond particles within the Co matrix, although non-uniform agglomerations were observed in all samples. This fact may be attributed to the surface tension of the diamond particles that cause aggregation among themselves [45]. For the upper Co deposit, the coating thickness was around 45 ± 3 µm. It can be assumed that the diamond particles dispersed in the deposition electrolyte are concomitantly deposited onto the substrate surface along with the cobalt ions.

**Figure 4:**
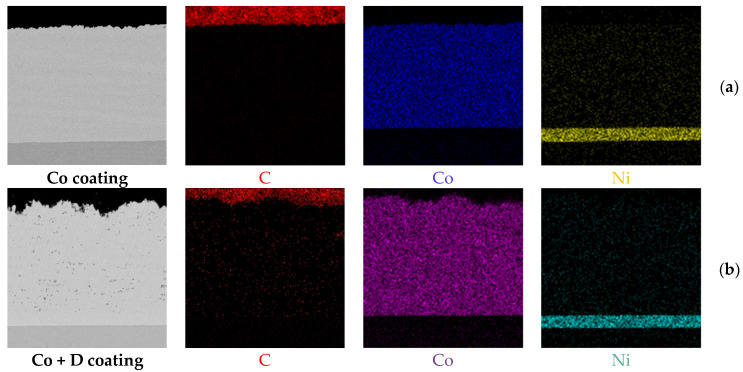
EDS elemental mapping analysis of the (**a**) Co coating and (**b**) Co + diamond particle composite coating.

EDS elemental mapping analysis, as shown in Figure 4, displays the distribution of chemical elements within the microstructures of the analyzed coatings in a cross-sectional view. The presence of Ni is in this case highlighted in both situations. The spectra also confirm the uniform distribution of diamond particles embedded within the Co deposit, indicating the successful incorporation of the reinforcing phase into the coating matrix.

### 3.2. Corrosion Behavior

The corrosion resistance of the Co-based coatings was assessed through electrochemical measurements conducted in a 3.5% NaCl solution at room temperature and compared to the S235 steel substrate. Before the investigations, the OCP values were determined within 30 min of monitoring. The OCP values become significantly more positive when the Co coating was present on the steel substrate material.

**Figure 5:**
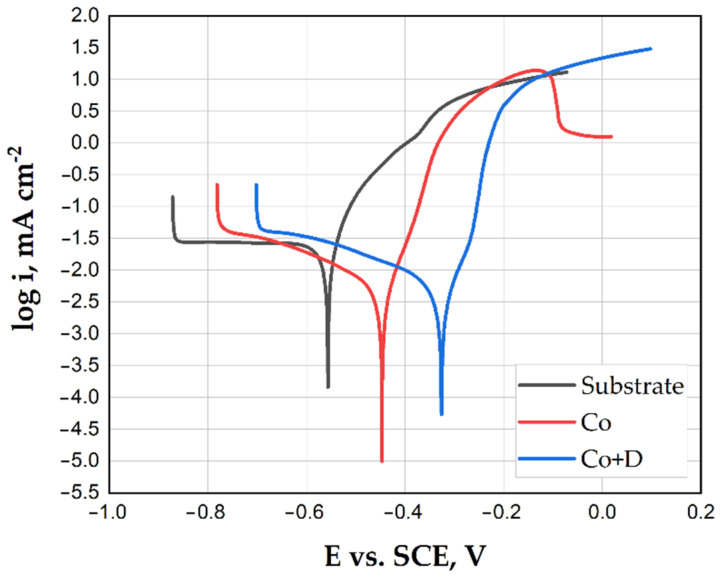
Corrosion behavior of the S235 substrate material, Co coating, and Co + diamond particle composite coating in 3.5 wt.% NaCl electrolyte and normal temperature, scan rate 0.16 mV s^−1^.

Figure 5 represents the polarization curves in the semilogarithmic form, as described by the Tafel extrapolation method. The Tafel plot represents the relationship between the measured potential and the logarithmic current density. It is obtained from the linear polarization curve, using the logarithmic function applied to the current density values. From the depicted polarization curves, the most relevant corrosion parameters were determined, like the corrosion potential (*E_corr_*) and corrosion current density (*i_corr_*); they are presented in Table 3. Analyzing the corrosion parameters while comparing the polarization curves in Figure 5 leads to the conclusion that the deposition of Co-based coatings has positively influenced the corrosion behavior of the investigated substrate, since the current density decreased ten orders of magnitude, from the initial value of 23.21 µA cm^−2^ for the steel, to 2.41 µA cm^−2^ for the Co coating and 3.82 µA cm^−2^ for the Co + D coating, correlated with the lower corrosion rates presented in Table 3. The addition of diamond particles did not bring further improvement in the corrosion resistance of the Co-based coatings, even if the constituent grains became coarser. Similarly, the surface roughness became higher, and the presence of the diamond particles in the coating created more active sites for corrosion initiation, but the corrosion rate remained similar to the pure Co coating. At more positive potential values (around −0.1 V vs. SCE), the Co-based coating presented a passivation tendency, which in the case of Co + D coatings was not observed. Moreover, the corrosion potential was shifted towards less negative values, indicating that the surface of the coating becomes more noble when diamond particles are incorporated into the cobalt electrodeposited layer in chloride-rich environment.

### 3.3. Hardness and Sliding Wear Resistance

The wear resistance and mechanical properties of the coatings are critical in extending the lifespan of engineering materials exposed to harsh operating conditions. In general, higher hardness improves sliding wear resistance, particularly in abrasive and adhesive wear scenarios. However, the effectiveness of this relationship depends on material properties, wear partners, environmental factors, and the nature of the wear mechanism involved [35,45]. Microhardness assessments demonstrate that both pure cobalt and composite coatings exhibit significantly higher hardness compared to the steel substrate. The average measured microhardness values for the substrate, Co coating, and Co + diamond particle composite coating were 129 ± 4 HV0.3, 327 ± 3 HV0.3, and 470 ± 7 HV0.3, respectively. Notably, the presence of hard diamond particles in the composite coating enhanced the resistance to local elastic deformation and significantly improved the overall hardness compared with that of pure cobalt coating, this aspect being attributed mainly to dispersion strengthening and particle strengthening effects.

**Figure 6:**
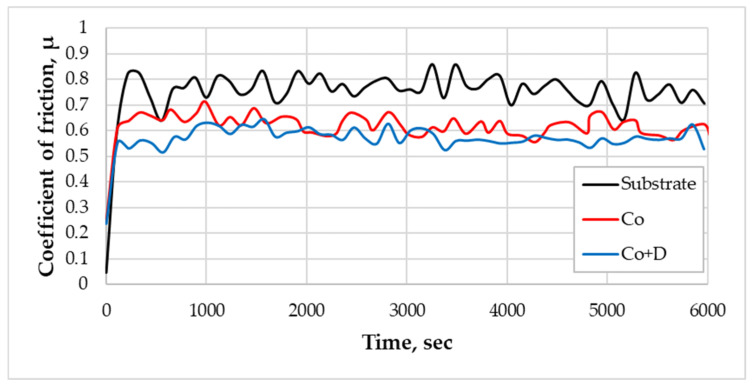
The evolution of the COF for the substrate, Co, and Co + D in dry sliding wear conditions and ambient temperature.

The wear resistance of both Co-based coatings and the substrate was assessed using the ball-on-disk method. During the test, the coefficient of friction (COF) between the sample and a 100Cr6 steel ball was continuously recorded. The variation of COF over time is depicted in Figure 6, while the corresponding numerical values are summarized in Table 4.

**Figure 7:**
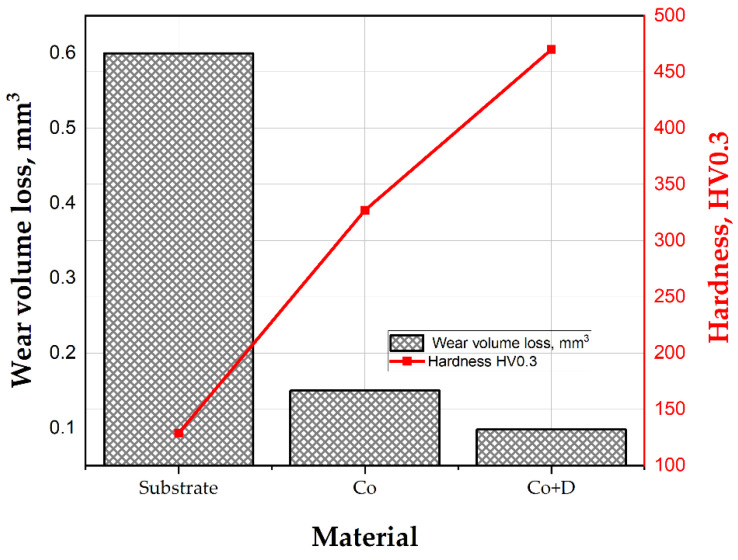
Wear volume loss during the sliding wear test and microhardness of the substrate, Co and Co + D coating.

The results indicate that the substrate exhibited a higher average COF (*µ*_averg_ = 0.751) compared to the Co-based coatings (*µ*_averg_ = 0.612/0.568), the lower values being recorded in the case of the composite coating containing diamond particles. The differences in COF and hardness significantly influenced the volume of the material lost during the sliding wear test (Figure 7). The presence of diamond particles incorporated in the more soft Co matrix enhanced its strength and minimized the direct contact between surfaces, leading to a reduction in friction. Remarkably, higher hardness values combined with a lower coefficient of friction resulted in reduced material loss, indicating improved sliding wear resistance. The Co composite coatings reinforced with diamond particles exhibited the lowest material loss, demonstrating the best wear resistance among all the tested materials. One can conclude that the inclusion of diamond particles enhances the hardness of the composite coating, leading to improved wear properties.

## 4. Conclusions

Cobalt and cobalt + diamond composite coatings were successfully fabricated using a one-step electrodeposition process on the surface of an S235 low-alloy steel. The coatings were morphologically analyzed and evaluated in regard to their morphology, corrosion resistance, and mechanical properties, including hardness and sliding wear resistance.

Microstructural investigations revealed that pure cobalt coatings exhibited a dense, nodular morphology, whereas the incorporation of diamond particles led to the formation of more elongated grains with a uniform dispersion of reinforcing particles. This structural modification contributed to enhanced mechanical performance. Hardness measurements confirmed that both cobalt-based coatings exhibited significantly higher hardness compared to the S235 steel substrate, with values of 327 ± 3 HV0.3 for pure cobalt coatings and 470 ± 7 HV0.3 for Co-diamond composite coatings, in contrast to 129 ± 4 HV0.3 for the uncoated steel. Notably, the addition of diamond particles did not alter the chemical properties of the composite coatings (Corr. rate 0.058 mm/year for pure Co vs. 0.058 mm/year for the Co + D coating), suggesting that the electrodeposition process effectively integrated the reinforcing phase without introducing defects or compromising the corrosion behavior, providing superior protection in a chloride environment compared to the uncoated steel substrate (Corr. rate of 0.283 mm/year). Tribological evaluation showed that the material loss decreased from 0.6 mm^3^ for the steel substrate to 0.15 mm^3^ (for Co) and 0.09 mm^3^ (for Co + D composite coatings), the latter one indicating the best wear resistance.

## Figures and Tables

**Table 1:** Chemical composition of the substrate material.

Steel	C [%]	Mn [%]	P [%]	S [%]	N [%]	Cu [%]	Fe [%]
S235	<0.17	<1.4	<0.035	<0.035	<0.12	<0.55	Bal.

**Table 2:** The chemical composition of the Ni and Co electrolyte and the deposition conditions.

Type of Coating	Electrolyte	Deposition Conditions
Ni interlayer	125 g L^−1^ NiSO_4_ · 7H_2_O + 25 g L^−1^ NiCl_2_ · 6 H_2_O + 20 g L^−1^ H_3_BO_3_ pH = 4.5–5	50 mA cm^−2^ for 5 min, at 50 °C, using a 99.99% nickel soluble anode
Co coating	300 g L^−1^ CoSO_4_· 6H_2_O + 50 g L^−1^ CoCl_2_ · 6H_2_O + 30 g L^−1^ H_3_BO_3_ pH = 4.5–5	50 mA cm^−2^ for 20 min, at 25 °C, using a graphite inert anode
Co + Diamond particles composite coating	300 g L^−1^ CoSO_4_· 6H_2_O + 50 g L^−1^ CoCl_2_ · 6H_2_O + 30 g L^−1^ H_3_BO_3_ + 2.5 g L^−1^ diamond particles (0.5 μm) pH = 4.5–5	25 mA cm^−2^ for 40 min, at 25 °C, using a graphite inert anode

**Table 3:** Corrosion parameters estimated from the Tafel extrapolation method.

Sample	*E* [mV] vs. Ag/AgCl	*i_corr_* [µA cm^−2^]	*Corr. rate* [mm Year^−1^]
S235 steel substrate (Substrate)	−556	23.2	0.283
Co-based coating (Co)	−446	2.4	0.058
Co + Diamond particles composite coating (Co + D)	−325	3.8	0.083

**Table 4:** Values of the recorded COF (*μ*) for the Co-based coatings compared to the S235 steel substrate.

Sample	*µ*_min_	*µ*_averg_	*µ*_max_
S235 steel substrate (Substrate)	0.096	0.751	0.859
Co-based coating (Co)	0.246	0.612	0.713
Co + Diamond particle composite coating (Co + D)	0.237	0.568	0.645

## Data Availability

The original contributions presented in this study are included in the article. Further inquiries can be directed to the corresponding authors.
